# Safety and necessity of omitting mediastinal lymph node dissection in cN0/N1 non-small cell lung cancer after neoadjuvant immunotherapy

**DOI:** 10.3389/fimmu.2025.1587658

**Published:** 2025-04-29

**Authors:** Yuheng Zhou, Wenyu Zhai, Weizhen Sun, Yongping Han, Zhichao Lin, Dihan Liu, Yan Zheng, Xiaojuan Luo, Zerui Zhao, Shoucheng Feng, Yaobin Lin, Hailin Tang, Hao Long

**Affiliations:** ^1^ Department of Thoracic Surgery, State Key Laboratory of Oncology in South China, Collaborative Innovation Center for Cancer Medicine, Sun Yat-sen University Cancer Center, Guangzhou, Guangdong, China; ^2^ Lung Cancer Research Center, Sun Yat-Sen University, Guangzhou, Guangdong, China; ^3^ Department of Thoracic Surgery, Yiyang Central Hospital, Yiyang, Hunan, China; ^4^ Department of Thoracic Surgery, Jiangmen Central Hospital, Jiangmen, Guangdong, China; ^5^ Department of Thoracic Surgery, Henan Cancer Hospital, Zhengzhou, Henan, China; ^6^ Department of Pathology, Yiyang Central Hospital, Yiyang, Hunan, China; ^7^ State Key Laboratory of Oncology in South China, Collaborative Innovation Center for Cancer Medicine, Sun Yat-sen University Cancer Center, Guangzhou, Guangdong, China

**Keywords:** non-small cell lung cancer, perioperative immunotherapy, tumor-draining lymph node, lymph node dissection optimization, T cell exhaustion

## Abstract

**Background:**

Lymph nodes are crucial for perioperative immunotherapy but have to be completely resected in surgery. Trials evaluating the safety and necessity of omitting systemic mediastinal lymph node (mLN) dissection in non-small cell lung cancer (NSCLC) are still absent.

**Methods:**

cN0/N1 NSCLC patients who received neoadjuvant immunotherapy and radical surgery were retrospectively collected from three institutions. Restricted cubic spline regression and receiver operating characteristic curve were used to analyze the association between mLN dissection number and survival outcomes. Confounding factors between selective and systemic mLN dissection groups were adjusted by inverse probability of treatment weighting (IPTW). The characteristics of memory CD8^+^ T cells in immunotherapy-treated mLN were identified by single-cell RNA and T-cell receptor sequencing (scRNA/TCR-seq) data retrieved from GSE185206.

**Results:**

From 2019 to 2021, 131 neoadjuvant-treated cN0/N1 NSCLC patients were collected. The mLN clearance rate was 98.5% in the whole cohort and 100% in patients with radiologically confirmed complete response. Resected lymph node counts were irrelevant with local recurrence, distant metastasis, or death. Compared with selective mLN dissection, systemic mLN dissection did not show any survival benefit but showed slightly higher postoperative recurrence risk in both unadjusted and IPTW-adjusted cohorts. scRNA/TCR-seq showed that stem-like exhausted CD8^+^ memory T cells were the progenitors of tumor-specific CD8^+^ T lymphocytes in primary tumors and were abundantly enriched in resected mLN.

**Conclusions:**

Omitting systemic mLN dissection was safe in cN0/N1 NSCLC patients who received neoadjuvant immunotherapy. Excessive mLN dissection may disrupt the repertoire of stem-like exhausted CD8^+^ memory T cells and consequently impair the efficacy of adjuvant immunotherapy.

## 1 Introduction

Systemic mediastinal lymph node (LN) dissection is currently the standard procedure in radical surgery for non-small cell lung cancer (NSCLC) (1). However, in the era of immunotherapy, excessive lymph node dissection not only increases the incidences of surgery-related complications, such as chylothorax and recurrent laryngeal nerve injury (2), but also destroys the intact lymphatic structures that are supposed to be the pivotal organs for adjuvant immunotherapy (3). According to the theory of the cancer-immunity cycle, tumor-draining lymph nodes (tdLNs) provide crucial places for dendritic cells and other antigen-presenting cells to activate naïve CD8^+^ T cells and therefore differentiate into progenitor and precursor exhausted T cells, which are the ancestors of tumor-specific T cells that finally infiltrate into the tumor (4, 5). A retrospective cohort study conducted by Liang et al. has indicated that over-dissection of mediastinal lymph nodes in lung cancer surgery is associated with poorer efficacy of immunotherapy when those patients face recurrence or metastasis (6). Unfortunately, considering that the accuracy of the radiologic diagnosis of mediastinal lymph node metastasis is still insufficient, and the lymphatic drainage patterns of lung cancer are not yet clear, little evidence supports that systemic lymph node dissection can be safely exempted, and the criteria of lymph node dissection number basically lack feasibility in clinical practice (7).

However, the unexpected efficacy of neoadjuvant/perioperative immunotherapy in non-small cell lung cancer revives the discussion of the lymph node dissection strategy (8–10). On the one hand, neoadjuvant treatment dramatically improves the clearance rate of primary tumors and lymph nodes (11, 12). On the other hand, the addition of adjuvant treatment further emphasizes the pivotal role of mediastinal lymph nodes in immunotherapy (13, 14).

To help address these gaps, we retrospectively established a cohort of cN0/N1 NSCLC patients who received neoadjuvant immunochemotherapy (nICT) and reported the negative rate of dissected lymph nodes, the diagnostic accuracy of pretreatment radiology in lymph node metastasis, and long-term follow-up data. We performed single-cell RNA and T-cell receptor sequencing (scRNA/TCR-seq) analyses to explore the characteristics of immunotherapy-treated lymph nodes and their association with adjuvant treatment effectiveness. Overall, this study manifested the safety and necessity of omitting mediastinal lymph node dissection in cN0/N1 NSCLC patients who received neoadjuvant immunotherapy.

## 2 Materials and methods

### 2.1 Patient cohort

**Figure 1:**
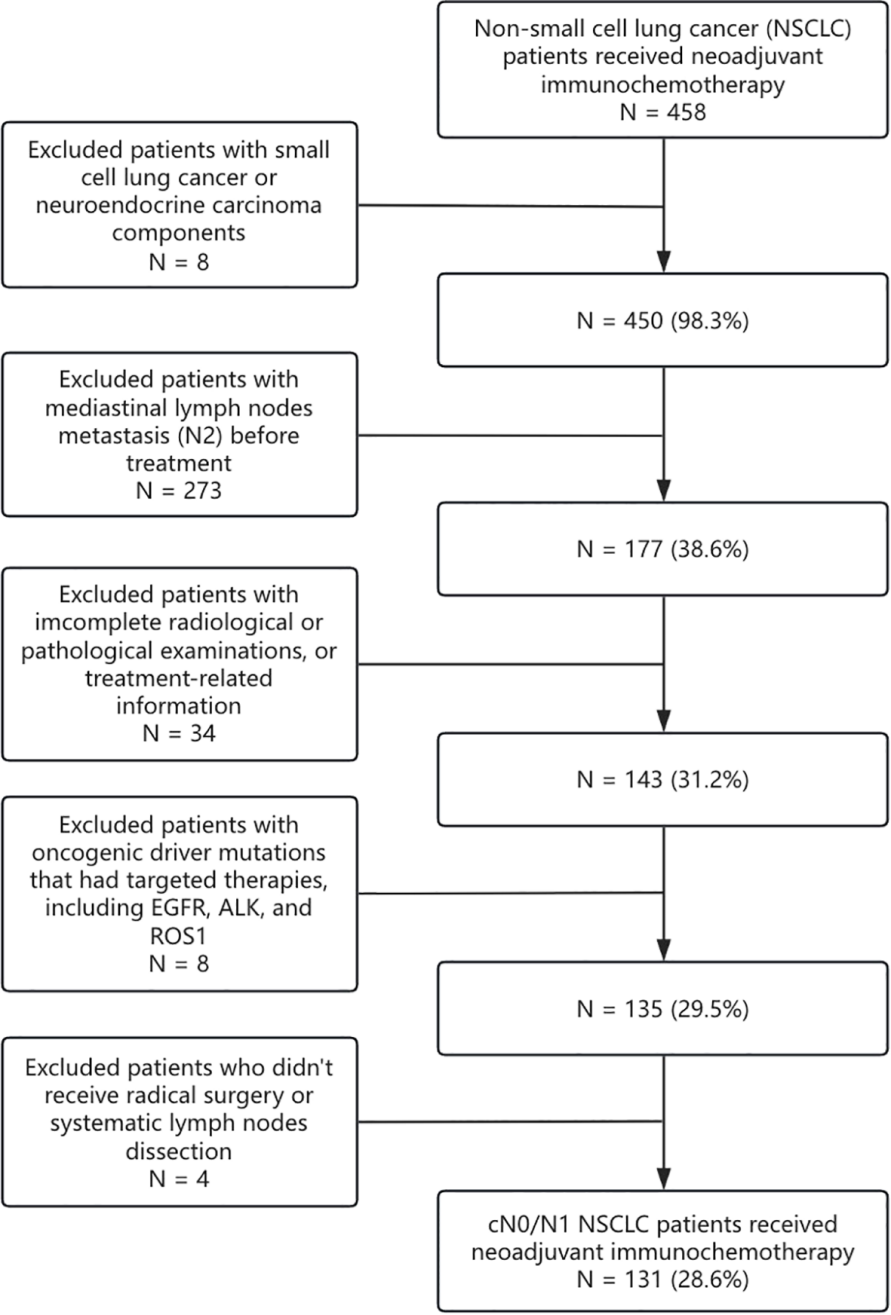
Trial flow diagram. NSCLC, non-small cell lung cancer; SCLC, small cell lung cancer; NEC, neuroendocrine carcinoma; EGFR, epidermal growth factor receptor.

From March 2019 to May 2021, a total of 131 cN0/N1 NSCLC patients who received nICT and radical surgery were collected from three medical centers (Sun Yat-sen University Cancer Center, Guangzhou; Jiangmen Central Hospital, Jiangmen; and Henan Cancer Hospital, Zhengzhou) (**Figure 1**). This study was approved by the institutional review boards from all participating centers (G2024-017-01). The requirement of informed consent was waived for the reason that no intervention was conducted. The study protocol was pre-registered on the ClinicalTrials (NCT06292052). The key eligibility criteria included 1) biopsy-proven non-small cell lung cancer, without small cell lung cancer components (15); 2) clinically staged as T1-4N0-1M0 according to American Joint Committee on Cancer/Tumor-Node-Metastasis (AJCC/TNM) 8th Edition (16); 3) received neoadjuvant immune checkpoint inhibitors and platinum-based doublet chemotherapy with no less than 2 cycles; 4) received radical surgery and systemic (≥3 N1 stations and ≥3 N2 stations) or passively selective lymph node dissection; 5) postoperative pathologic assessment complied with the International Association for the Study of Lung Cancer (IASLC) multidisciplinary recommendations (17); and 6) without known EGFR, ALK, or ROS1 mutation. Demographic, clinical, radiologic, and pathologic data were retrospectively retrieved from the electronic medical record system.

### 2.2 Pretreatment and preoperative clinical staging

The pretreatment clinical staging was assessed using 18-fluorodeoxyglucose-positron emission tomography (PET) or contrast-enhanced computed tomography (CT) within 2 weeks before the first neoadjuvant treatment (18). The T staging was determined by the maximum diameter or the invasion extent of the primary tumor measured by radiologic examinations. Endobronchial ultrasound-guided transbronchial needle aspiration (EBUS-TBNA) and mediastinoscopy were used for the invasive assessment of abnormal lymph nodes (19). Furthermore, lymph nodes with the shortest diameter larger than 10 mm or elevated uptake of 2-deoxy-2-fluoro-d-glucose and with abnormal radiologic features were also defined as metastasis. The N staging is determined by the combination of pathologic and radiologic examinations. Magnetic resonance imaging (MRI) of the brain was necessary for patients who did not undergo PET/CT examinations. The preoperative clinical staging was assessed in the same way after the completion of neoadjuvant treatment and within 2 weeks before the operation.

### 2.3 Outcome assessment

The surgical resection specimens were completely sampled for primary tumors with 3 cm or less in maximum diameter or cut across in 0.5-cm-thick serial sections for larger sizes. The resected lymph nodes were also fully assessed with every cross-section. Complete pathologic response (pCR) is defined as no viable tumor cells observed in pathology slides of all resected tissues, and such outcome would be staged as ypT0N0 according to the AJCC/TNM 8th edition (16, 17). The definition of major pathologic response (MPR) is a significant reduction of all histologic types of lung cancer with a clinical cutoff of 10%. Those who experienced asymmetric treatment response, such as having complete regression in primary tumor but residual tumor cells in lymph nodes, were also regarded as MPR. Lung cancer biomarker testing and radiology examinations were regularly adopted in postoperative follow-up. The last follow-up date was February 1, 2025. Event-free survival (EFS) and overall survival (OS) were calculated from cycle 1, day 1 of neoadjuvant treatment to the last follow-up. Follow-up data were censored at 3 years. Recurrence was confirmed by imaging and/or pathology and further classified as local or distant recurrence according to the metastatic extent defined by the thoracic cavity.

### 2.4 Statistical analysis

Inverse probability of treatment weighting (IPTW) was conducted between the selective lymph node dissection group and the systemic lymph node dissection group (20). Continuous variables with normal distribution were presented by mean and standard deviation (SD) and analyzed by Student’s *t*-tests. The others with skewed distribution were described by median and interquartile range (IQR) and analyzed by Mann–Whitney *U* tests. Categorical variables were demonstrated by frequency and percentage and analyzed by chi-square or Fisher’s exact tests according to the estimated frequency. EFS and OS status were demonstrated using the Kaplan–Meier curves, and the differences between subgroups were analyzed by long-rank tests (21). Univariable and multivariable logistic regression and Cox regression analyses were conducted to discriminate risk factors (22). Receiver operating characteristic (ROC) analysis and restricted cubic spline (RCS) method were also used to explore the association between lymph node dissection number and follow-up outcomes (23, 24). All of the statistical analyses were performed by SPSS version 27.0 (Armonk, NY, USA) and the R Statistical Software (v4.3.2; R Core Team 2022). Type I error was set as 0.05 (two-sided).

### 2.5 scRNA/TCR-seq analysis

scRNA/TCR data from Pai JA et al. were retrieved from Gene Expression Omnibus (GEO) (GSE185206), including 18 postoperative samples of primary tumor and paired mediastinal lymph nodes (25). scRNA-seq datasets were log10-normalized individually and integrated using the Seurat (v5.2.1) and Harmony (v1.2.3) packages (26, 27). Mitochondria, ribosome, and TCR genes were excluded to avoid cluster bias. Uniform Manifold Approximation and Projection (UMAP) with the first 50 principal components were used for dimensionality reduction (28). K-nearest neighbor graph was constructed, and the Louvain algorithm was used to identify phenotypic clusters with a resolution of 1 (29). The enrichment and pattern categorization of the TCR clone were analyzed using the scRepertoire (v1) package.

## 3 Results

### 3.1 Patient characteristics

A total of 131 NSCLC patients staged as cT1-4N0-1M0 and received nICT followed by radical surgery were retrospectively reviewed from three institutions. The baseline characteristics are shown in **Table 1**. Of those, 23.7% (31/131) were staged as cN0, and 76.3% (100/131) were staged as cN1. Of those staged as N, 43.5% (57/131) were determined by PET/CT, and others were by CT. Of 131 patients, 12.2% (16) received invasive assessment of mediastinal lymph nodes. Of 131, 63.4% (83) received immunotherapy-based adjuvant treatment. Specifically, seven (5.3%) patients staged as IB received nICT due to involvement of the main bronchus and intolerable pneumonectomy or sleeve resection. The median number of resected N1 and N2 stations lymph nodes was respectively 8 (interquartile range, 5 to 12) and 10 (interquartile range, 5 to 16). The pCR and MPR rates were respectively 47.3% (62/131) and 62.6% (82/131). With a median follow-up of 24.7 months [95% CI, 18.8–30.6], the 3-year EFS and OS were 90.6% and 96.2%, respectively.

**Table 1:** Baseline characteristics of the whole cohort in SYSUCC, JMCH, and HNCH.

Characteristics	Level	SYSUCC	JMCH	HNCH
		N = 71	N = 31	N = 29
Age, no. (%)	<65	47 (66.2)	20 (64.5)	18 (62.1)
	≥65	24 (33.8)	11 (35.5)	11 (37.9)
Sex, no. (%)	Male	58 (81.7)	28 (90.3)	28 (96.6)
	Female	13 (18.3)	3 (9.7)	1 (3.4)
Smoking history, no. (%)	Never	18 (25.4)	5 (16.1)	6 (20.7)
	F/C	53 (74.6)	26 (83.9)	23 (79.3)
ICI types, no. (%)	PD-1	68 (95.8)	28 (90.3)	29 (100.0)
	PD-L1	3 (4.2)	3 (9.7)	0 (0.0)
Neoadjuvant treatment cycle, no. (%)	≤3	61 (85.9)	23 (74.2)	25 (86.2)
	>3	10 (14.1)	8 (25.8)	4 (13.8)
Clinical stage, no. (%)	IB	4.97 (1.92)	4.92 (2.02)	4.21 (1.54)
	IIA	3 (4.2)	2 (6.5)	1 (3.4)
	IIB	0 (0.0)	1 (3.2)	2 (6.9)
	IIIA	37 (52.1)	15 (48.4)	19 (65.5)
Clinical N stage, no. (%)	N0	31 (43.7)	13 (41.9)	7 (24.1)
	N1	41 (57.7)	19 (61.3)	15 (51.7)
Radiology exam types, no. (%)	CT	30 (42.3)	12 (38.7)	14 (48.3)
	PET/CT	57 (80.3)	21 (67.7)	21 (72.4)
Tumor histology, no. (%)	LUSC	14 (19.7)	10 (32.3)	8 (27.6)
	Non-LUSC	23 (32.4)	14 (45.2)	11 (37.9)
Adjuvant treatment status, no. (%)	No	48 (67.6)	17 (54.8)	18 (62.1)
	Yes	47 (66.2)	20 (64.5)	18 (62.1)

SYSUCC, Sun Yat-sen University Cancer Center; JMCH, Jiangmen Central Hospital; HNCH, Henan Cancer Hospital; ICI, immune checkpoint inhibitor; PD-1, programmed death-1; PD-L1, programmed death-L1; CT, computed tomography; PET, positron emission tomography; LUSC, lung squamous cell carcinoma; F/C, former/current.

### 3.2 Lymph node clearance outcomes

**Figure 2:**
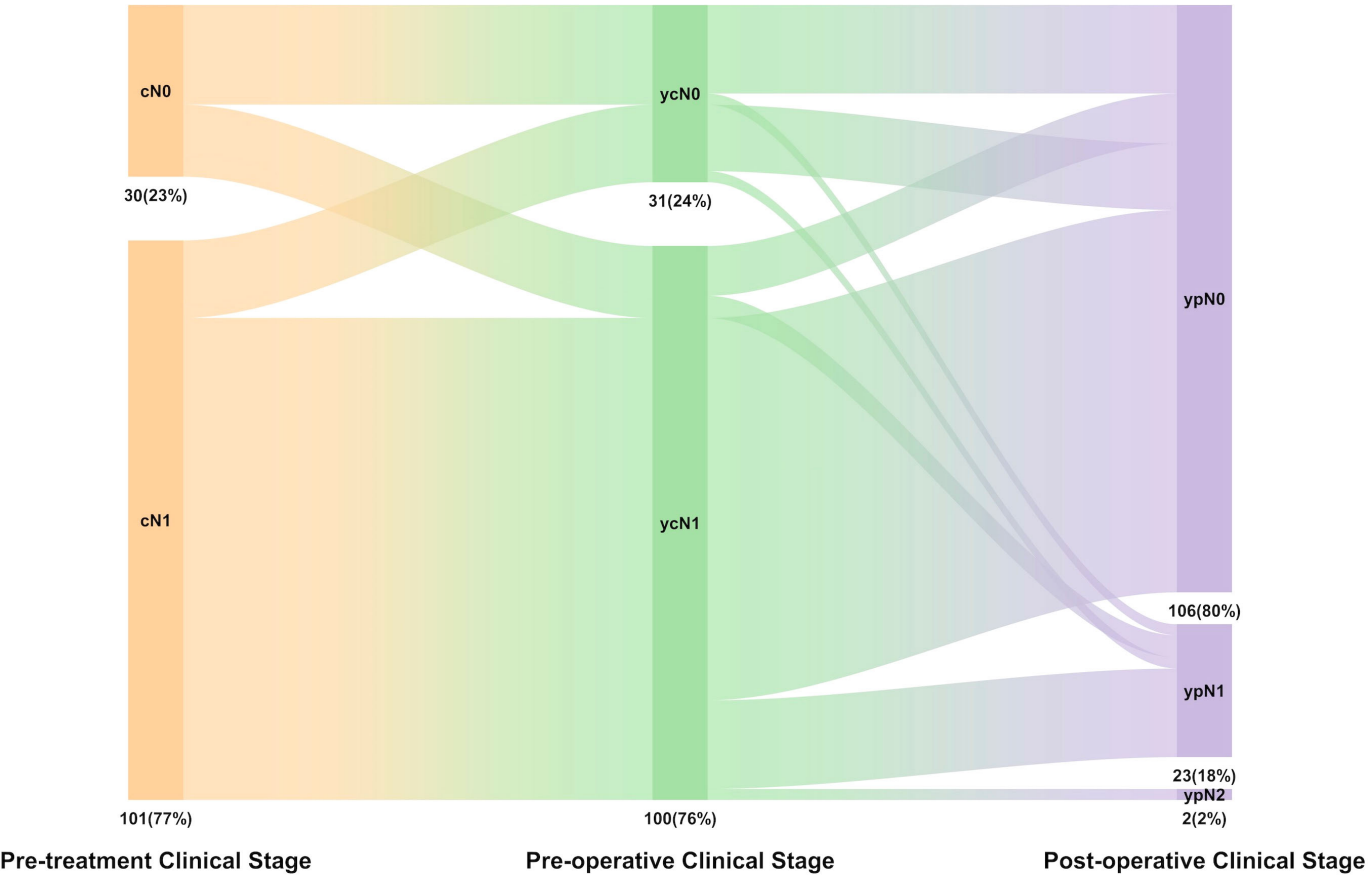
The distribution of N stage in pre-treatment clinical stage (cN), pro-operative clinical stage (ycN), pathologic stage (pN), and lymph node downstage relationship.

According to the postoperative pathology reports, the lymph node clearance rate (ypN0/N1) was 98.5% (129/131), including 80.9% ypN0 (106/131) and 17.6% ypN1 (23/131) (**Figure 2**). Among the ypN1 patients, two were hilar lymph node metastases (Station 10), and the other 21 were intralobar lymph node metastases (Stations 11–14). The multivariate and univariate logistic regression analyses showed that demographic characteristics (e.g., sex, age, and smoking history), clinical stages, and histological types were not risk factors for mediastinal lymph node clearance (ypN0/N1) or intralobar lymph node clearance (ypN0). Furthermore, according to the radiologic evaluation performed before the operation, radiologically confirmed complete response (R-CR) or partial response (R-PR) was found to have a significant association with lymph node clearance (ypN0) compared with that evaluated as stable disease (88% vs. 20%, OR = 0.03 [95% CI: 0.01–0.14], *p* < 0.001).

### 3.3 Recurrence patterns and relative risk factors

For the 10 patients who experienced recurrence or metastasis (R/M) during the follow-up period, one experienced bone metastasis, two experienced surgical site recurrence, three experienced non-regional lymph node recurrence, and four experienced intrapulmonary metastases. The Cox proportional hazards survival regression preliminarily showed that clinical characteristics (e.g., sex, age, and smoking history), neoadjuvant therapy procedures (e.g., types of immune checkpoint blockade inhibitors and cycles of neoadjuvant treatment), and pathologic characteristics (e.g., histological type) were not risk factors for EFS or OS, including the dissection number of N1 station or N2 station. Moreover, adjuvant treatment was not confirmed as a protective factor for R/M or death as well, whether in the pCR or non-pCR subgroup (**Table 2** and **Supplementary Table S1-2**).

**Table 2:** Multivariate analysis for event-free survival in cN0/N1 patients with NSCLC who underwent neoadjuvant immunotherapy.

Characteristics	Log(HR)	95% CI	*p*-Value
Age			
<65	—	—	
≥65	−0.98	−2.6, 0.61	0.227
ICI types			
PD-1	—	—	
PD-L1	0.89	−1.2, 3.0	0.412
Neoadjuvant treatment cycle			
≤3	—	—	
>3	−0.42	−2.5, 1.7	0.695
Baseline N staging			
cN0	—	—	
cN1	0.23	−1.4, 1.8	0.782
Tumor histology			
LUSC	—	—	
Non-LUSC	−0.37	−2.0, 1.2	0.648
Adjuvant treatment status			
No	—	—	
Yes	0.86	−0.71, 2.4	0.282
Resected lymph node count			
Resected N1+N2 LN count	−0.01	−0.14, 0.12	0.889
Resected N2 LN count	0.06	−0.11, 0.24	0.489

HR, hazard ratio; CI, confidence interval; ICI, immune checkpoint inhibitor; PD-1, programmed death-1; PD-L1, programmed death-L1; LUSC, lung squamous cell carcinoma.

Furthermore, in order to discriminate the potential risks of postoperative N1 station metastasis, we classified the ypN1 patients into intralobar ypN1 and hilar ypN1. The Cox regression analysis showed that patients with hilar ypN1 experienced similar risks of R/M (*p* = 0.98) or death (*p* = 0.98) compared to those with only intralobar lymph nodes involved. Furthermore, the difference in R/M or death risk between patients with multiple and single ypN1 station metastases was also not observed (*p* = 0.99).

### 3.4 Association between lymph node dissection number and follow-up outcomes

**Figure 3:**
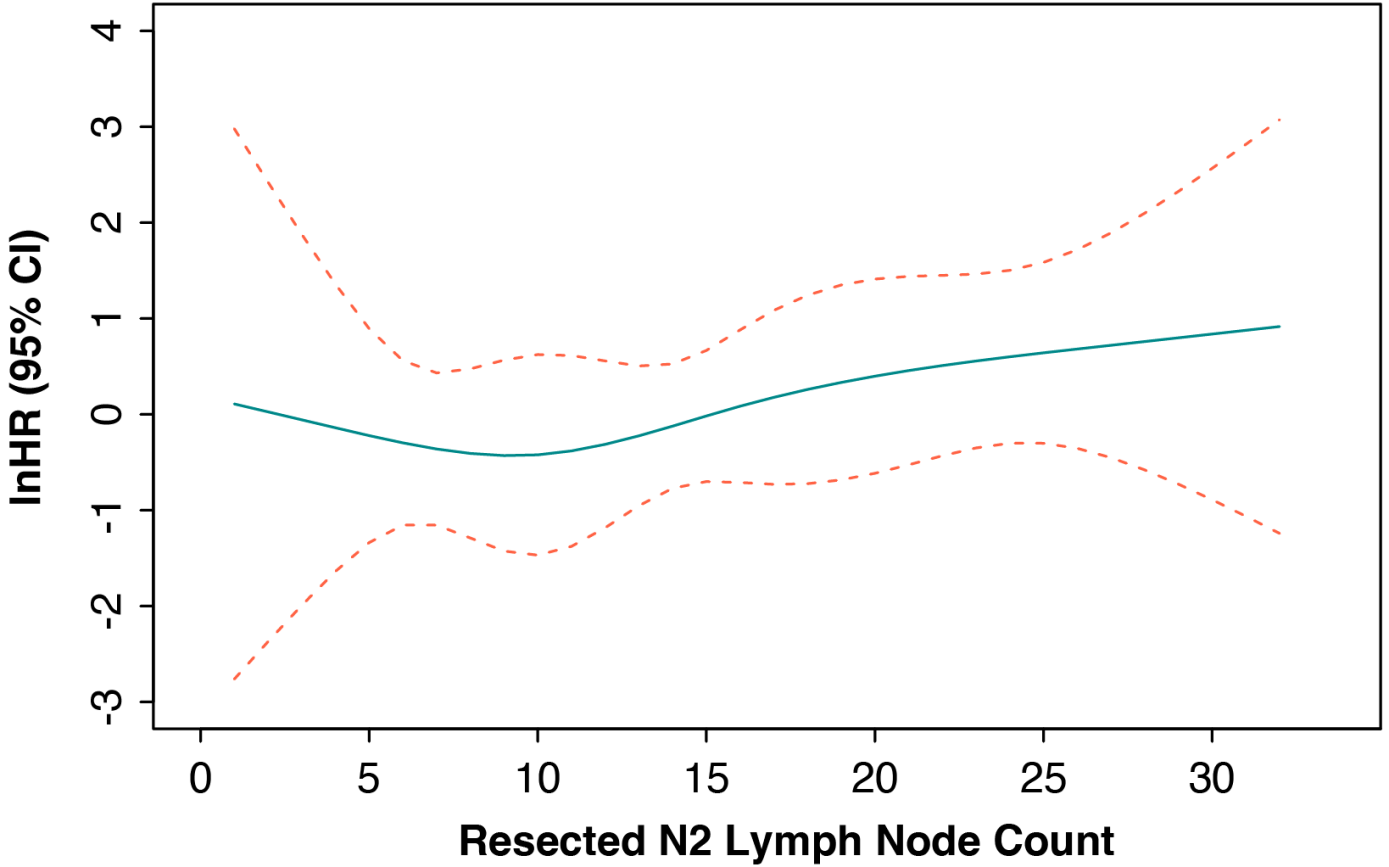
Cox-adjusted hazards ratio of event-free survival in the whole cohort. HR, hazard ratio; CI, confidence interval.

We further applied the RCS method in the Cox regression model to demonstrate the continued hazard ratio (HR) values between the count of resected N2 lymph nodes and EFS outcomes. The results showed that the HR values were all close to 1 and that no significant relationship was observed (**Figure 3**). Moreover, the ROC analysis also found that no optimized N2 lymph node resection number could be found to predict the EFS or OS outcome (**Figures S1A, B**).

**Figure 4:**
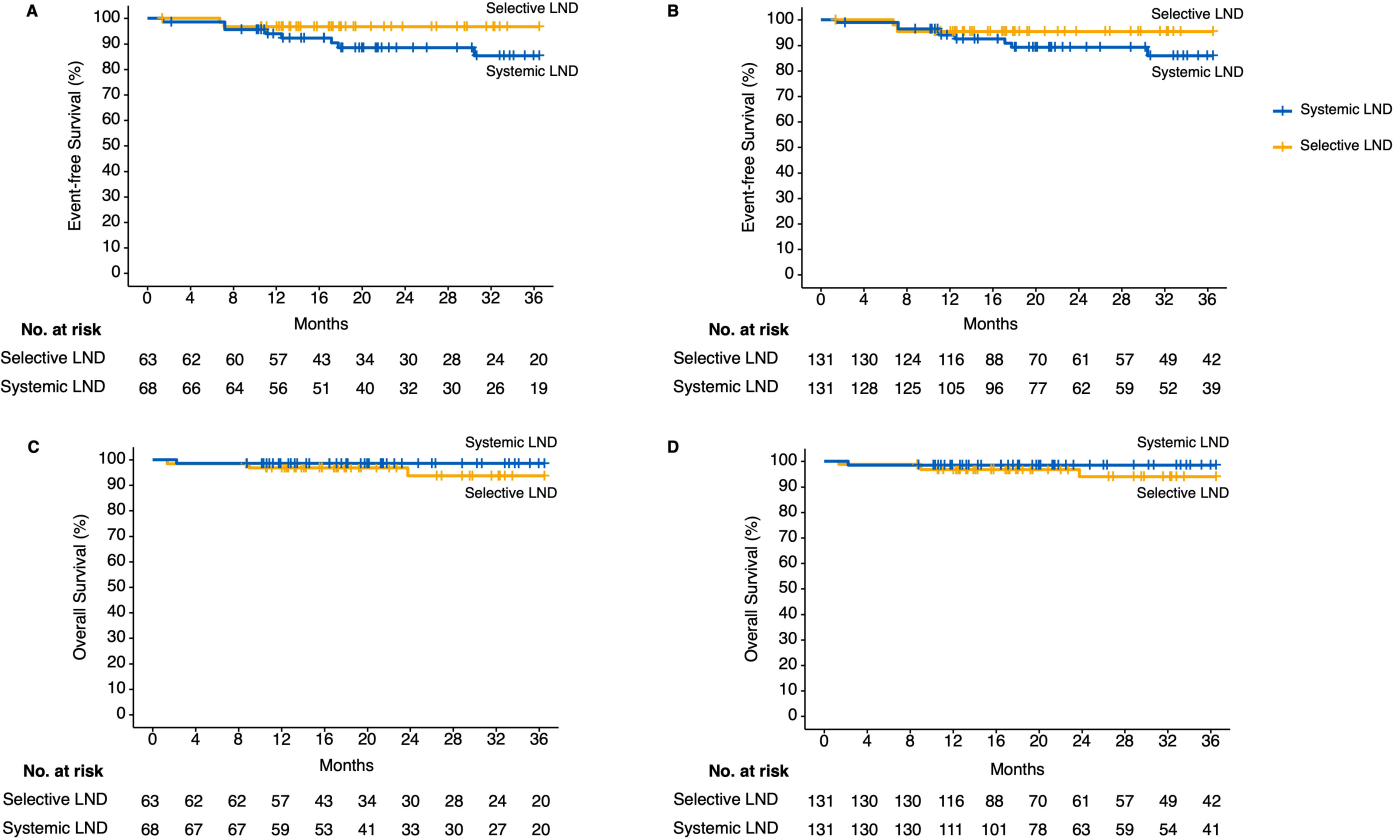
Kaplan–Meier estimates of follow-up outcomes among the patients in the unadjusted and inverse probability of treatment weighting (IPTW)-adjusted cohort grouped by selective or systemic lymph node dissection. **(A)** Three-year event-free survival outcomes in unadjusted cohort. **(B)** Three-year event-free survival outcomes in IPTW-adjusted cohort. **(C)** Three-year overall survival outcomes in unadjusted cohort. **(D)** Three-year overall survival outcomes in IPTW-adjusted cohort. IPTW, inverse probability of treatment weighting; LND, lymph node dissection.

In addition, combining with the RCS-calculated structural break point for the HR values of EFS and previously reported suggestions, we separated patients who had less than or equal to 12 N2 station lymph nodes dissected into non-systemic lymph node dissection group and the other into systemic lymph node dissection group. To balance the confounding clinicopathologic characteristics between the two groups, we performed IPTW using gender, age (divided by 65 years old), maximum diameter of baseline tumor (millimeter), histology (squamous cell carcinoma or not), cycles of neoadjuvant immunotherapy (divided by 3 times), pathologic response (pCR or not), and history of adjuvant immunotherapy. The standardized mean difference (SMD) of baseline characteristics after IPTW adjustment were all smaller than 0.1 (**Supplementary Table S3**). The 3-year EFS and OS showed no statistical significance between the systemic lymph node dissection group and non-systemic lymph node dissection group in the unadjusted (3-year EFS: 85.3% vs. 96.8%, HR = 3.6 [95% CI, 1.05–12.53], *p* = 0.08; 3-year OS: 98.5% vs. 93.7%, HR = 0.31 [95% CI, 0.04–2.19], *p* = 0.28) and IPTW-adjusted cohorts (3-year EFS: 86% vs. 95.9%, HR = 3.6 [95% CI, 1.05–12.53], *p* = 0.08; 3-year OS: 98.15% vs. 93.5%, HR = 0.31 [95% CI, 0.04–2.19], *p* = 0.28) (**Figures 4A–D**). Although no statistical outcome was reached, patients in the non-systemic lymph node group showed a trend of reduced R/M instead.

### 3.5 Stem-like exhausted CD8^+^ T cells were enriched in post-neoadjuvant treatment lymph nodes

**Figure 5:**
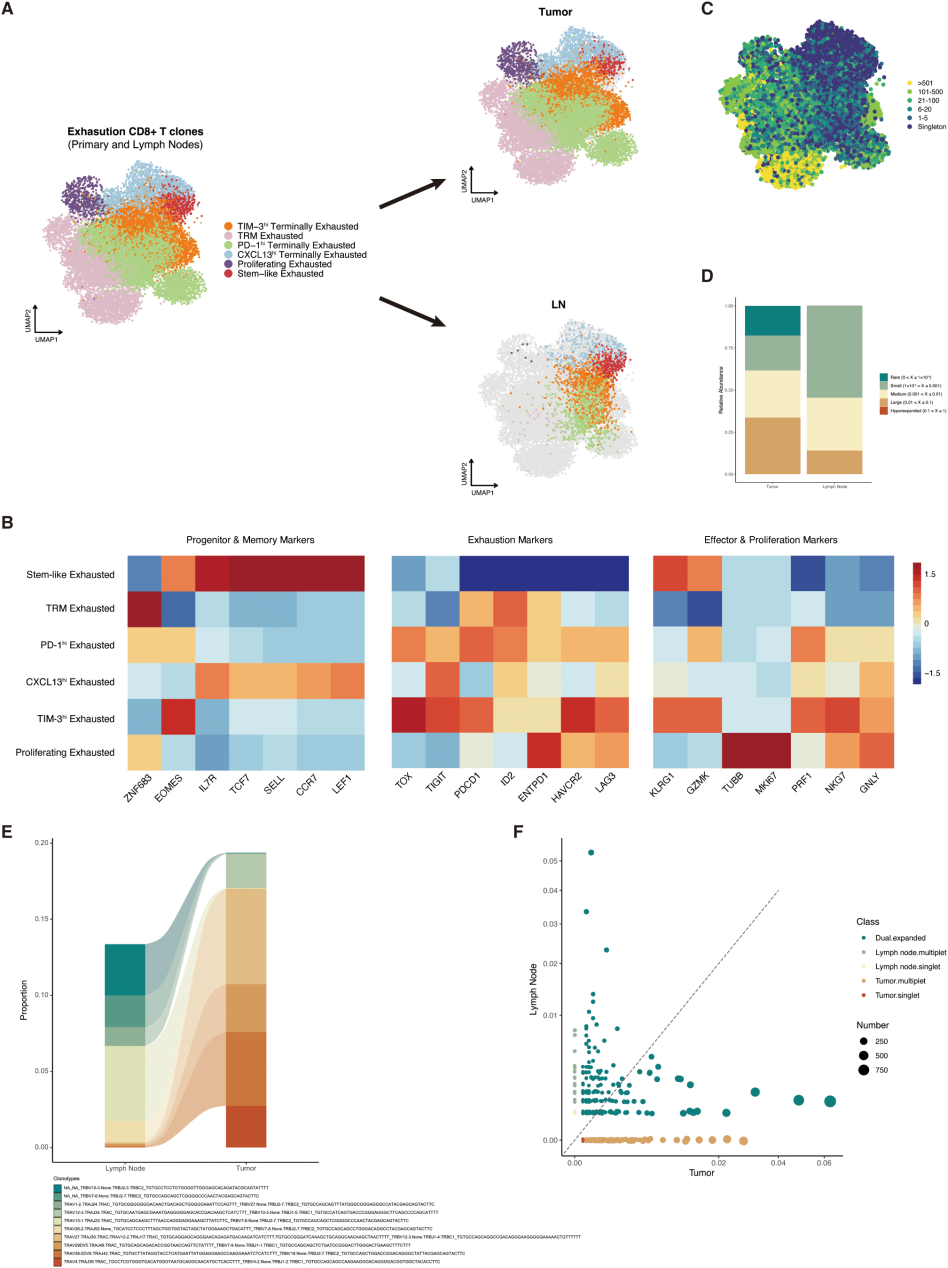
Characteristics and distribution of stem-like CD8^+^ T cells in primary tumors and draining lymph nodes. **(A)** Uniform Manifold Approximation and Projection (UMAP) plot of CD8^+^ clusters split by tumor (upper) and lymph nodes (lower). **(B)** Expression level heat map of progenitor, memory, exhaustion, effector, and proliferation markers among the CD8^+^ clusters. **(C)** UMAP overlaid with TCR_αβ_ clone size. **(D)** Relative abundance of TCR_αβ_ clone size in tumor and lymph nodes. **(E)** Alluvial plot showing the expansion characteristics of TCR_αβ_ between tumor and lymph nodes. **(F)** Scatter plot showing the distribution of singlet, multiple, and dual TCR expansion characteristics in tumor and lymph nodes. TCR, T-cell receptor; UMAP, Uniform Manifold Approximation and Projection; TRM, tissue-resident memory.

Previous studies have indicated that stem-like exhausted CD8^+^ T cells, which express both memory-related and exhaustion-related markers, were concentrated in the draining lymph nodes and were the progenitor of tumor-specific CD8^+^ T cells (25, 30). We then tried to distinguish those subtypes in the resected lymph nodes from patients who received immunotherapy and analyzed their potential functions in adjuvant immunotherapy. In this study, we collected the scRNA/TCR-seq datasets of 16 primary tumor samples and two paired lymph node samples from NSCLC patients after immune checkpoint blockade (ICB) from GEO (GSE185206). CD8^+^ T cells that exhibited high expression levels of exhaustion gene (*HAVCR2*, *ENTPD1*, *LAG3*, *PDCD1*, *CXCL13*, *TOX*, and *GZMB*) in the primary tumor were extracted and clustered. The CD8^+^ T cells in the draining lymph nodes that had to share TCR sequences were further integrated. These CD8^+^ T cells were finally divided into six clusters, including stem-like exhausted T cells, tissue-resident memory T cells, proliferating T cells, and three terminally exhausted populations labeled by characteristic exhaustion gene (*PDCD1*, *CXCL13*, and *HAVCR2/TIM-3*) (**Figure 5A**). Similar to reported gene sets, memory-like features (such as *TCF7*, *SELL*, *LEF1*, and *CCR7*, *IL7R*) and effector-like features (such as *GZMK* and *KLRG1*) were both selectively expressed in stem-like clusters (**Figure 5B**). The UMAP plot split by tissue (tumor or lymph nodes) distinctly demonstrated that stem-like exhausted CD8^+^ T cells were mainly distributed in resected lymph nodes, while terminally exhausted CD8^+^ T cells were in the primary tumor.

By pairing the TCR information with cluster phenotypes, we found that TCR clones were mostly expanded in those terminally exhausted clusters and tissue-resident memory T cells, which was in accordance with the assumptive development trajectory (**Figure 5C**). The proportion of TCR relative abundance in tumor and lymph nodes also showed that large-expanded TCR clones were more commonly observed in tumor samples (**Figure 5D**). We then defined the TCR sequences with identical VDJC gene names and CDR3 nucleotide as the same clone and then described the changes of the same TCR clonotype expansion level between tumors and lymph nodes. As we expected, the TCR trajectory showed that most of the tumor-specific CD8^+^ T cells were expanded from the memory T cells in mediastinal lymph nodes (**Figure 5E** **).** The scatter plot also demonstrated that tumor-specific CD8^+^ T cells that highly expanded in primary tumor sites mostly shared TCR clones of memory T cells (dual expanded clonotypes) in mediastinal lymph nodes (**Figure 5F**).

## 4 Discussion

This multicenter study retrospectively established a cohort of cN0/N1 NSCLC patients who received neoadjuvant immunotherapy and radical surgery. By reporting the landscape of lymph node clearance and analyzing the association between the extent of mediastinal lymph node dissection and survival outcomes, we first demonstrated the safety of omitting systemic mediastinal lymph node dissection in neoadjuvant-treated NSCLC patients who had uninvolved mediastinal lymph node at baseline. Furthermore, to demonstrate the necessity of preserving intact mediastinal lymph nodes, we analyzed the scRNA/TCR-seq data of paired tumor and lymph node samples retrieved from NSCLC patients treated with anti-PD-1 inhibitors. Finally, we proposed the residence of stem-like CD8^+^ memory T cells in ICB-treated lymph nodes and their potential impact on adjuvant immunotherapy and disease recurrence. Overall, this study for the first time comprehensively illustrated the safety and necessity of omitting mediastinal lymph node dissection in nICT-treated cN0/N1 NSCLC patients.

The balance between the extent of lymph node dissection and surgical benefits is a frequently discussed dilemma. The thorough lymph node dissection indeed removes micrometastasis and provides sufficient samples for pathologic assessment, but the surgical-related adverse effects such as unintended injuries to vessels, nerves, or other adjacent tissues are also significantly increased, especially in complicated operations such as lung and esophageal surgeries. In the era of immunotherapy, the contradiction is further intensified when facing NSCLC patients who received neoadjuvant immunotherapy. On the one side, immunotherapy-based neoadjuvant treatment prominently improves the complete pathologic response rate of both primary tumors and lymph nodes. On the other side, fibrosis and tissue adhesion caused by neoadjuvant treatment predominantly increase the risk of surgery to a higher degree. However, it is noteworthy that the significant regression of lymph nodes also eradicates the micrometastasis and therefore creates favorable conditions for omitting systemic mediastinal lymph node dissection, especially for patients with uninvolved mediastinal lymph nodes at baseline.

In our study, we showed that the true negative rate of mediastinal lymph nodes in cN0/N1 NSCLC patients after receiving nICT reached 98.5%, and those patients that had radiologically confirmed complete response at the time before surgery showed 100% mediastinal lymph node clearance, which preliminarily proved the safety of omitting systemic lymph node dissection. We further included various lymph node characteristics such as resected lymph node counts and N1 metastatic station counts to analyze their association with recurrence and death. Considering that the hilar and intralobar (N1 station) lymph node dissection number is irrelevant to surgical procedures and is highly correlated with post-operative anatomy, we generally took the mediastinal (N2 station) lymph node dissection number into consideration. However, the Cox-adjusted HR values of EFS showed that there was no optimized N2 lymph node dissection number, and a linear or non-linear relationship was not observed, which indicated that patients with more N2 lymph node dissection did not experience EFS benefits. Concerning that insufficient lymph node dissection may leave occult lymph node metastasis and cause regional recurrence or distant metastasis consequently, this study further compared the EFS and OS outcomes between patients who received selective mediastinal lymph node dissection due to tissue fibrosis and adhesion and systemic mediastinal lymph node dissection with a relatively long follow-up period. As expected, we found that selective lymph node dissection was not associated with increased events of recurrence, metastasis, or death in both unadjusted and IPTW-adjusted cohorts. To our surprise, although the OS outcomes between the two groups remained similar, patients with systemic lymph node dissection showed a higher risk of recurrence, which potentially indicated the importance of an intact lymph node system in the postoperative period. Actually, a similar analysis has been conducted in the population of NSCLC patients who received surgery alone and without neoadjuvant treatment. Ling et al. proposed that inadequate or excessive lymph node dissection could disrupt long-term survival (31). Recently, another study reported that resected lymph nodes greater than 16 were associated with poorer efficacy of postoperative immunotherapy in those populations (6). However, considering that these studies only enrolled patients who did not undergo immunotherapy before surgery, the differences among the conclusions further indicated the uniqueness and specialty of lymph nodes activated by immunotherapy-based treatment and the significance of this novel research in nICT-treated NSCLC patients.

In addition to proving the safety of omitting systemic lymph node dissection, we noticed that preserving intact mediastinal lymph nodes may also improve survival outcomes. Although adjuvant immunotherapy is regarded as a standard part of perioperative treatment, its efficacy is still being challenged. A few studies indicated that the removal of draining lymph nodes (dLNs), which is a crucial organ for the function of anti-PD-1/PD-L1 inhibitors, is the key point for the unsatisfactory efficacy. During the neoadjuvant period, a majority of naïve CD8^+^ T cells are activated and proliferated in the dLN. *In vitro* experiments removing dLN before receiving ICB inhibitors already show extremely poor regression of the primary tumor (32, 33), while *in situ* injection of anti-PD-L1 inhibitors to the dLN demonstrates similar efficacy compared with intravenous injection. Therefore, the excessive dissection of negative lymph nodes could also destroy the intact immune system and finally disrupt the efficacy of adjuvant immunotherapy. In this study, the cross-tissue integration analysis of paired tumor and lymph node scRNA/TCR-seq data showed that the activated tumor-cytotoxic CD8^+^ T cells were mainly differentiated from progenitor-exhausted CD8^+^ T cells. In addition, the TCR trajectory demonstrated that the stem-like exhausted CD8^+^ T cells were abundantly inhabited in resected dLN and sequentially developed into terminally exhausted CD8^+^ T cells and migrated to primary tumor sites. Feature analysis further showed that stem-like CD8^+^ T cells were highly expressed memory cell genes, which represented their roles in responding to adjuvant immunotherapy and long-term anti-tumor functions.

This study had several limitations. As a multicenter study, although multiple statistical methods such as covariate regression and IPTW adjustment were conducted, the inherent defect of confounding bias was still inevitable. Furthermore, to confirm the sustainable role of differentiating tumor-specific CD8^+^ T cells, the scRNA/TCR-seq should also be conducted in peripheral blood during the adjuvant treatment period (9).

## 5 Conclusion

In conclusion, for cN0/N1 NSCLC patients who received nICT, systemic mediastinal lymph node dissection was not associated with survival benefits but disrupted the repertoire of stem-like tumor-cytotoxic CD8^+^ T cells. Depending on the mediastinal lymph node clearance efficacy of nICT, omitting systemic lymph node dissection in cN0/N1 NSCLC patients is safe and necessary. Prospective clinical trials evaluating the non-inferiority of selective mediastinal lymph node dissection should be further conducted.

## Data Availability

The data presented in the study are deposited in the figshare repository, accession number: doi.org/10.6084/m9.figshare.28862972.v1; doi.org/10.6084/m9.figshare.28862399.v1; doi.org/10.6084/m9.figshare.28855505.v1.

## Supplementary material

Supplementary Figure 1 Receiver operating characteristics analysis of the association between N2 station lymph node dissection count and event-free or overall survival outcomes. ROC, Receiver operating characteristics; EFS, Event-free survival; OS, Overall-survival; HR, Hazard ratio; CI, Confidence interval.
