# Supplementary figures and images for: Safety and necessity of omitting mediastinal lymph node dissection in cN0/N1 non-small cell lung cancer after neoadjuvant immunotherapy
Source: Front Immunol. 2025 Apr 29;16:1587658. doi: 10.3389/fimmu.2025.1587658 (PMC12069321; doi:10.3389/fimmu.2025.1587658)

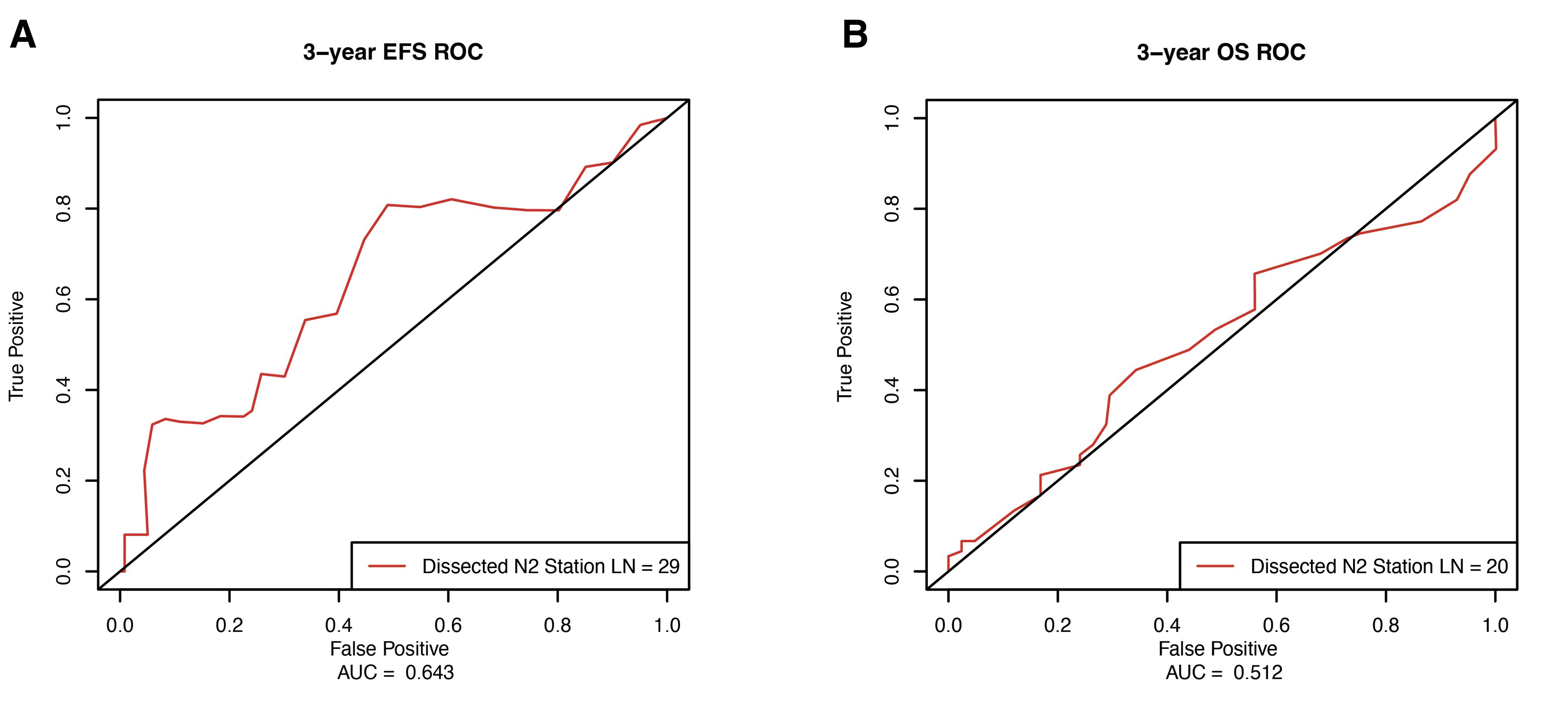

Supplement: Supplementary Figure 1 — Receiver operating characteristics analysis of the association between N2 station lymph node dissection count and event-free or overall survival outcomes. ROC, Receiver operating characteristics; EFS, Event-free survival; OS, Overall-survival; HR, Hazard ratio; CI, Confidence interval. [file Image1.jpeg]
