# Supplementary material for: Safety and necessity of omitting mediastinal lymph node dissection in cN0/N1 non-small cell lung cancer after neoadjuvant immunotherapy
Source: Front Immunol. 2025 Apr 29;16:1587658. doi: 10.3389/fimmu.2025.1587658 (PMC12069321; doi:10.3389/fimmu.2025.1587658)
Supplement: Supplementary file 2 [file Table1.docx]

**Supplemental Tables**

**Table S1.** Multivariate analysis for event-free survival in the pCR and non-pCR subgroups of cN0/N1 patients with NSCLC underwent neoadjuvant immunotherapy

| **Characteristics** | **log(HR)** | **95% CI** | ***p* value** |
| --- | --- | --- | --- |
| **pCR Cohort** |  |  |  |
| Adjuvant Treatment Status |  |  |  |
| No | — | — |  |
| Yes | 0.29 | -2.6, 3.2 | 0.842 |
| Resected N1+N2 LN Count | 0.27 | -0.08, 0.61 | 0.126 |
| Resected N2 LN Count | 0.00 | -0.32, 0.32 | 0.988 |
| **Non-pCR Cohort** |  |  |  |
| Adjuvant Treatment Status |  |  |  |
| No | — | — |  |
| Yes | 0.90 | -1.2, 3.0 | 0.403 |
| Resected N1+N2 LN Count | -0.11 | -0.29, 0.07 | 0.211 |
| Resected N2 LN Count | 0.14 | -0.09, 0.37 | 0.238 |

HR, Hazard Ratio; CI, Confidence Interval; pCR, Complete Pathologic Response; LN, Lymph Node

**Table S2.** Multivariate analysis for overall survival in cN0/N1 patients with NSCLC underwent neoadjuvant immunotherapy

| **Characteristics** | **log(HR)** | **95% CI** | ***p* value** |
| --- | --- | --- | --- |
| **Overall Cohort** |  |  |  |
| Adjuvant Treatment Status |  |  |  |
| No | — | — |  |
| Yes | -0.30 | -2.3, 1.7 | 0.768 |
| Resected N1+N2 LN Count | 0.02 | -0.21, 0.26 | 0.840 |
| Resected N2 LN Count | -0.16 | -0.52, 0.20 | 0.386 |
| **pCR Cohort** |  |  |  |
| Adjuvant Treatment Status |  |  |  |
| No | — | — |  |
| Yes | 0.83 | -2.4, 4.0 | 0.612 |
| Resected N1+N2 LN Count | 0.04 | -0.23, 0.32 | 0.749 |
| Resected N2 LN Count | -0.30 | -0.79, 0.18 | 0.219 |
| **Non-pCR Cohort** |  |  |  |
| Adjuvant Treatment Status |  |  |  |
| No | — | — |  |
| Yes | -1.2 | -4.0, 1.7 | 0.421 |
| Resected N1+N2 LN Count | -0.06 | -0.41, 0.29 | 0.746 |
| Resected N2 LN Count | -0.02 | -0.50, 0.45 | 0.926 |

HR, Hazard Ratio; CI, Confidence Interval; pCR, Complete Pathologic Response; LN, Lymph Node

**Table S3.** Baseline Characteristics of Inverse Probability Treatment Weighting Cohort

| **Characteristics** | **Level** | **Selective LND** | **Systemic LND** | ***p* value** | **SMD** |
| --- | --- | --- | --- | --- | --- |
|  |  | **N = 131** | **N = 131** |  |  |
| Age - no. (%) | <65 | 88 (67.3) | 88.8 (67.6) | 0.972 | 0.006 |
|  | ≥65 | 43 (32.7) | 42.6 (32.4) |  |  |
| Sex - no. (%) | Male | 114 (86.7) | 114.2 (86.9) | 0.984 | 0.004 |
|  | Female | 17 (13.3) | 17.3 (13.1) |  |  |
| Smoking History - no. (%) | No | 30 (22.9) | 29.2 (22.2) | 0.929 | 0.017 |
|  | F/C | 101 (77.1) | 102.3 (77.8) |  |  |
| ICIs Types - no. (%) | PD-1 | 123 (93.5) | 126.6 (96.3) | 0.485 | 0.13 |
|  | PD-L1 | 8 (6.5) | 4.8 (3.7) |  |  |
| Neoadjuvant Treatment Cycle - no. (%) | ≤3 | 110 (83.8) | 109.5 (83.3) | 0.949 | 0.012 |
|  | >3 | 21 (16.2) | 21.9 (16.7) |  |  |
| Baseline Tumor Diameter - mean (SD) |  | 4.84 (1.70) | 4.77 (2.11) | 0.867 | 0.033 |
| Clinical Stage - no. (%) | IB | 6 (4.7) | 7 (5.3) | 0.996 | 0.045 |
|  | IIA | 3 (2.2) | 2 (1.7) |  |  |
|  | IIB | 69 (52.3) | 70 (53.1) |  |  |
|  | IIIA | 53 (40.8) | 52 (39.9) |  |  |
| Radiology Exams Types - no. (%) | CT | 79 (60.1) | 74 (56.9) | 0.718 | 0.066 |
|  | PET/CT | 52 (39.9) | 57 (43.1) |  |  |
| Tumor Histology - no. (%) | LUSC | 100 (76.3) | 101 (76.9) | 0.944 | 0.013 |
|  | Non-LUSC | 31 (23.7) | 30 (23.1) |  |  |
| Adjuvant Treatment Status - no. (%) | No | 48 (36.3) | 49 (37.4) | 0.904 | 0.022 |
|  | Yes | 83 (63.7) | 82 (62.6) |  |  |

ICI, Immune Checkpoint Inhibitor; PD-1, Programmed Death-1; PD-L1, Programmed Death-L1; CT, Computed Tomography; PET, Positron Emission Tomography; LUSC, Lung Squamous Cell Carcinoma; LND, Lymph node dissection; SMD, Standarized mean difference; F/C, Former/Current
